# Supplementary material for: Sensing of cytosolic LPS through caspy2 pyrin domain mediates noncanonical inflammasome activation in zebrafish
Source: Nat Commun. 2018 Aug 3;9:3052. doi: 10.1038/s41467-018-04984-1 (PMC6076302; doi:10.1038/s41467-018-04984-1)
Supplement: Supplementary file 1 — Supplementary Information [file 41467_2018_4984_MOESM1_ESM.pdf]

Supplementary information

Sensing of cytosolic LPS through caspy2 pyrin domain mediates noncanonical inflammasome activation in zebrafish

Dahai Yang[1], Xin Zheng[1], Shouwen Chen[1], Zhuang Wang[1], Wenting Xu[1], Jinchao Tan[1], Tianjian Hu[1], Mingyu Hou[1], Wenhui Wang[1], Zhaoyan Gu[1], Qiyao Wang[1,2,3,4], Ruilin Zhang[4,5], Yuanxing Zhang[1,2,3,4], Qin Liu[1,2,3,4 *]

[1] State Key Laboratory of Bioreactor Engineering, East China University of Science and Technology, Shanghai 200237, China

[2] Laboratory for Marine Biology and Biotechnology, Qingdao National Laboratory for Marine Science and Technology, Qingdao 266071, China

[3] Shanghai Engineering Research Center of Marine Cultured Animal Vaccines, Shanghai 200237, China

[4] Shanghai Collaborative Innovation Center for Biomanufacturing, Shanghai 200237, China

[5] State Key Laboratory of Genetic Engineering, School of Life Sciences, Fudan University, Shanghai 200433, China

*Correspondence: qinliu@ecust.edu.cn

This PDF file contains 11 Supplementary Figures and 1 Supplementary Table.

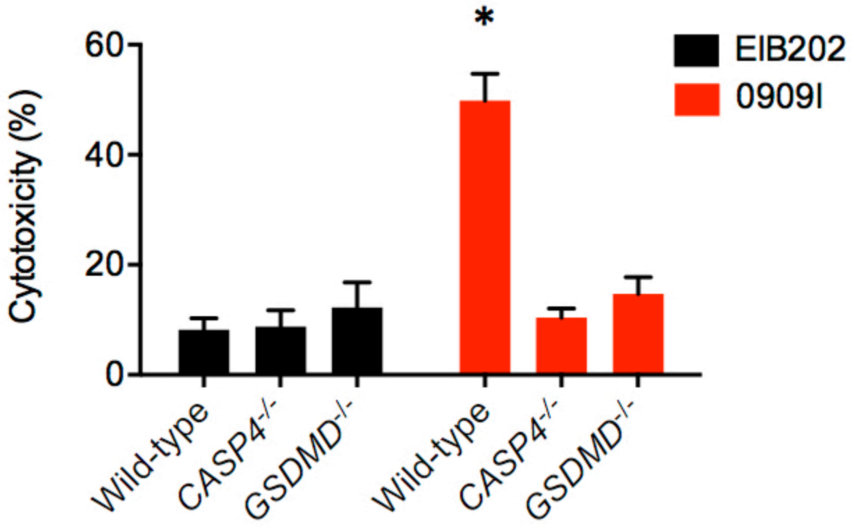

Supplementary Fig. 1. 0909I *E. piscicida* infection induces noncanonical inflammasome activation in HeLa cells. Wild-type, *CASP4*$^{-/-}$, and *GSDMD*$^{-/-}$ HeLa cells were infected with wild-type (EIB202) or 0909I *E. piscicida* for 2 h at a multiplicity of infection (MOI) of 50, or were left uninfected. The supernatants were then subjected to a lactate dehydrogenase (LDH) assay. Results are representative of at least three independent experiments, and error bars denote SD of triplicate wells. *$p < 0.05$ (*t*-test).

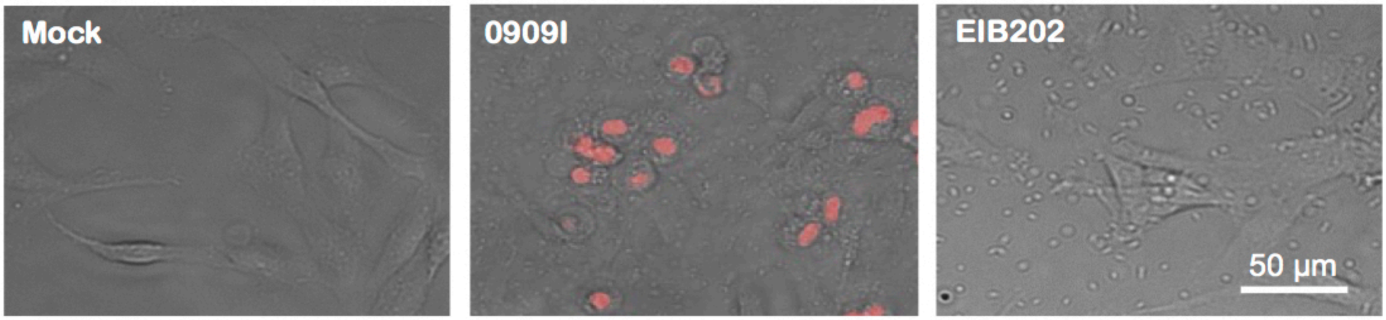

Supplementary Fig. 2. 0909I *E. piscicida* infection induces pyroptosis in ZF4 cells. Images of uninfected ZF4 zebrafish fibroblasts and those infected with wild-type (EIB202) or 0909I *E. piscicida* for 2 h at a multiplicity of infection (MOI) of 50. Propodium iodide (PI) was added to detect loss of plasma membrane integrity. Results are representative of at least three independent experiments. Scale bar, 50 µm.

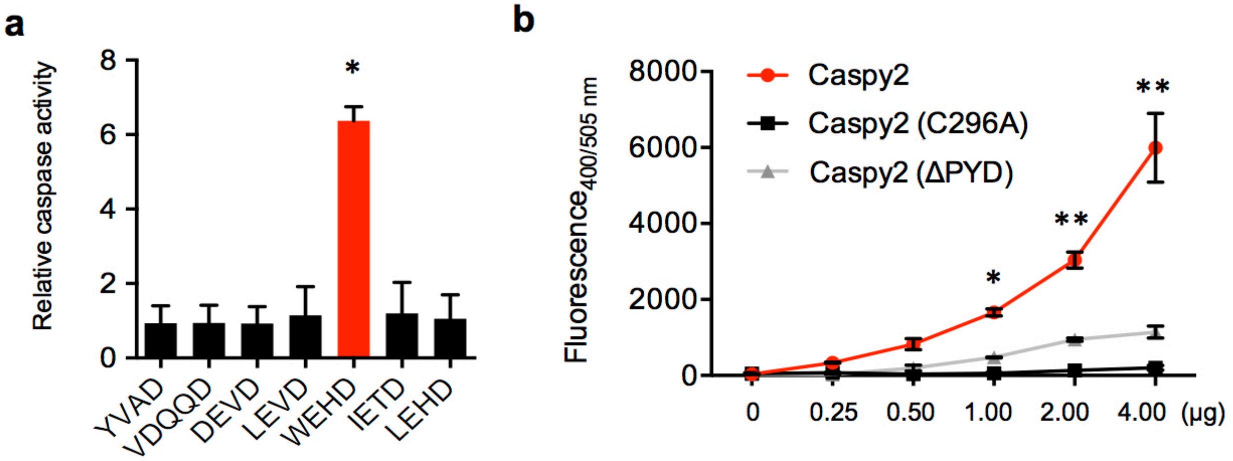

Supplementary Fig. 3. Caspase substrates activity of caspy2. (a) Relative caspase activity was measured by incubating purified HA-tagged caspy2 in transfected HEK293T cells with fluorogenic and chromogenic substrates of caspase-1 (YVAD), caspase-2 (VDQQD), caspase3/7 (DEVD), caspase-4 (LEVD), caspase-5 (WEHD), caspase-8 (IETD), and caspase-9 (LEHD). (b) Purified HA-tagged caspy2, caspy2 (C296A), and caspy2 ((ΔPYD) were subjected to a caspase-5 substrate (WEHD) cleavage assay. (a and b) Results are representative of at least two independent experiments, and error bars denote the SD of triplicate wells. *$p < 0.05$, **$p < 0.01$ (ANOVA).

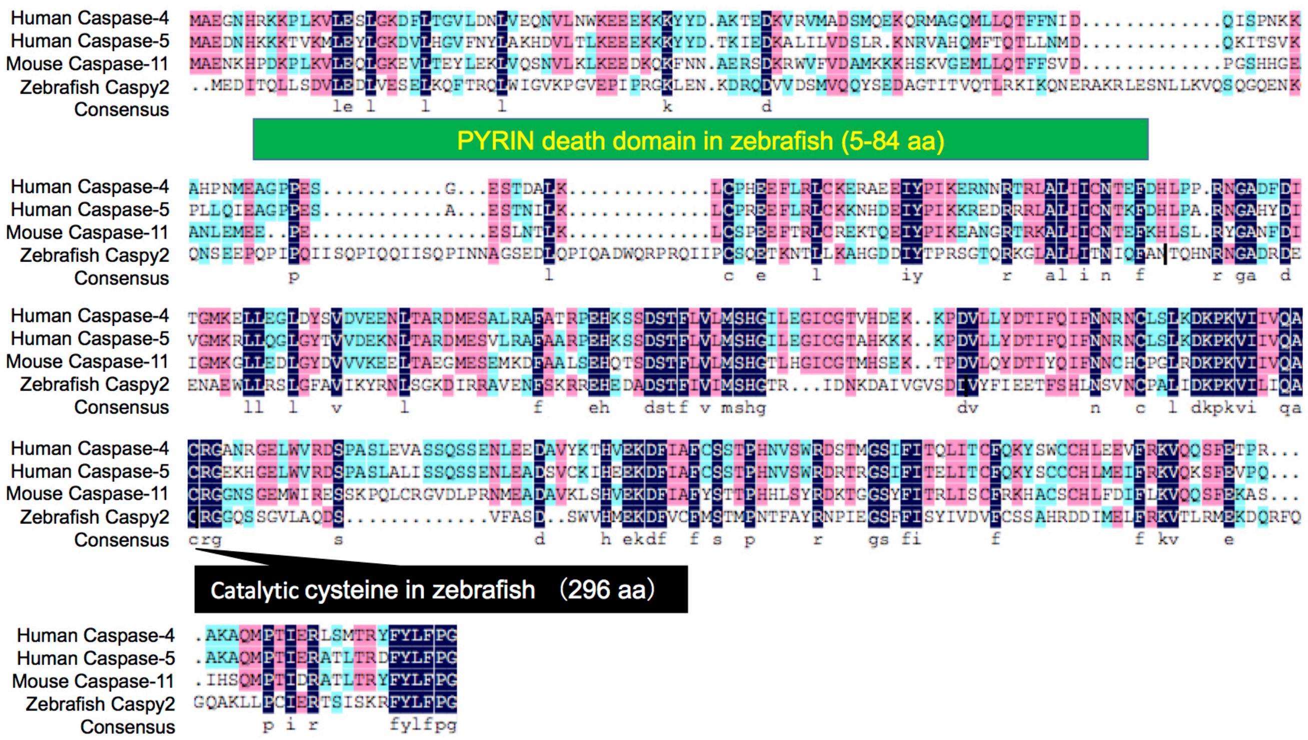

Supplementary Fig. 4. Alignment of the zebrafish caspy2 sequence with that of human caspase-4/5 and mouse caspase-11. Amino acid residues conserved across all four sequences are shaded dark blue. The green box indicates the pyrin death domain residues in zebrafish[10]. The black arrowhead box indicates the conserved caspase catalytic cysteine residue.

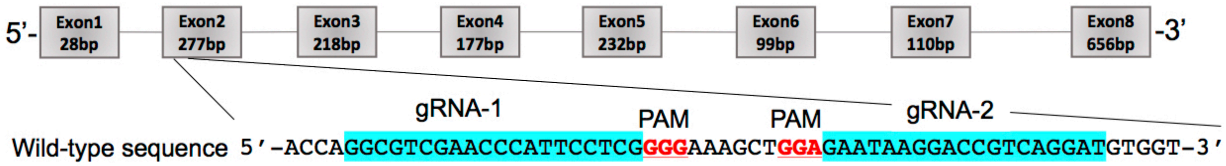

pSpCas9(BB)-*caspy2* gRNA-GFP (μg/2 x 10^5 cells)	KO-1 Efficiency (%)	KO-2 Efficiency (%)
0.25	10.55 ± 1.87	8.32 ± 2.13
0.50	26.81 ± 3.65	22.14 ± 5.32
0.75	7.42 ± 3.21	12.86 ± 2.75

Supplementary Fig. 5. Generation of c*aspy2*-KO ZF4 cells by CRISPR/Cas9-mediated genome editing. The upper diagram shows the *caspy2* region containing the targeted locus. The sequences targeted to generate the knockout (KO) lines used in experiments are shown below. The effect on KO efficiency of transfecting 2 × 10^5 ZF4 cells with different doses of pSpCas9(BB)-*caspy2* gRNA-GFP plasmids. Data shown are representative of three independent experiments.

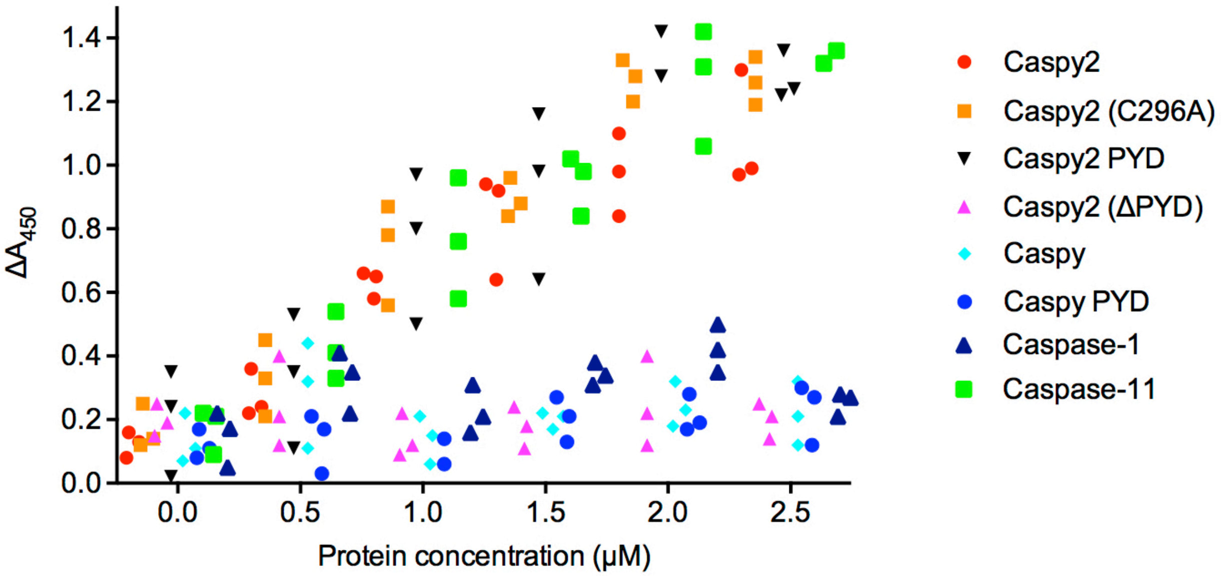

Supplementary Fig. 6. Caspy2 directly binds to lipopolysaccharide (LPS). HA-tagged caspy2, caspy2 (C296A), caspy2 (ΔPYD), caspy2 PYD, caspy, caspy PYD, caspase-1, and caspase-11 were immunoprecipitated from HEK293T cells overexpressing the proteins. The capacity of the indicated caspases to bind LPS was determined using an LPS-based enzyme-linked immunosorbent assay (ELISA).

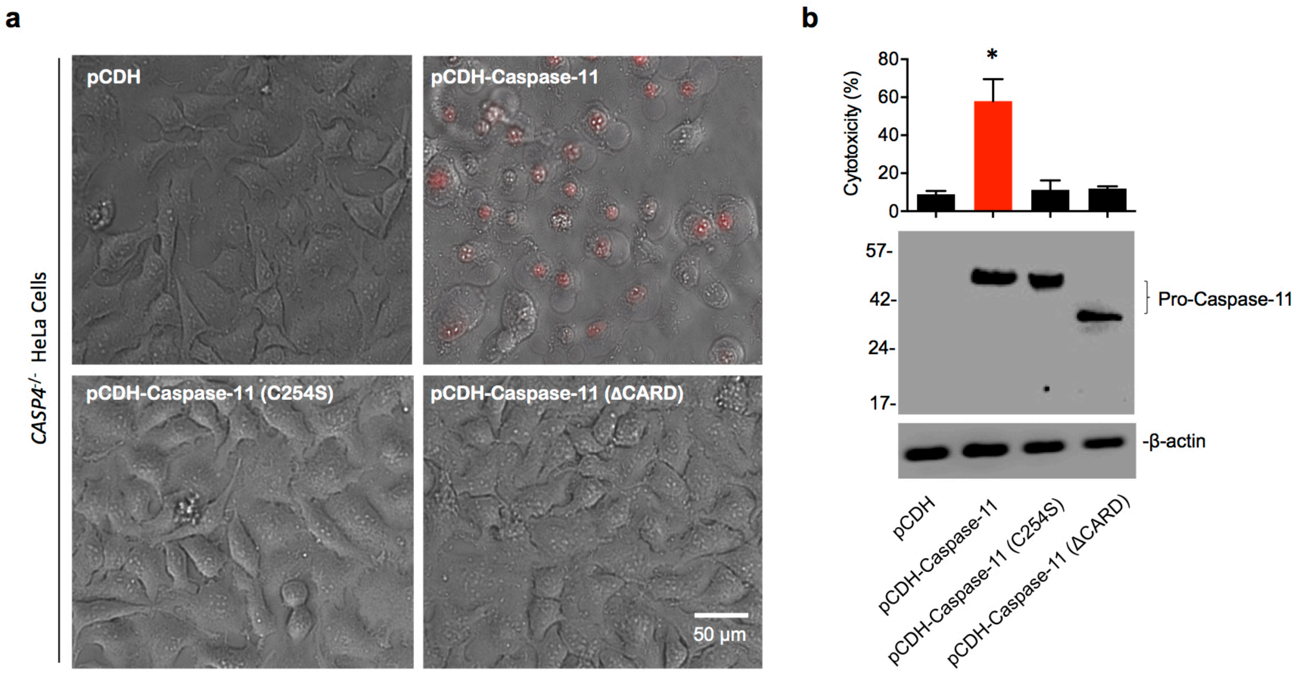

Supplementary Fig. 7. Caspase-11 gates intracellular LPS-mediated non-canonical inflammasome activation. (a and b) *CASP4*$^{-/-}$ HeLa cells were transduced with a vector expressing wild-type caspase-11, caspase-11 (C2254S), caspase-11 (ΔCARD), or the empty vector. Cells were primed with LPS for 4 h, before being stimulated with cholera toxin B subunit (CTB) plus LPS for 12 h. Images were taken as in Figure 1d (a). Propidium iodide (PI) was added to detect the loss of plasma membrane integrity. Arrows denote cells with pyroptotic-like features. Scale bar, 50 μm. Supernatants from the indicated HeLa cells were analyzed for cell death measured by lactate dehydrogenase (LDH) release, and immunoblotting for the caspase-11 forms indicated is shown (b). (a-b) Results are representative of at least three independent experiments, and error bars denote the SD of triplicate wells. * $p < 0.05$ (*t*-test).

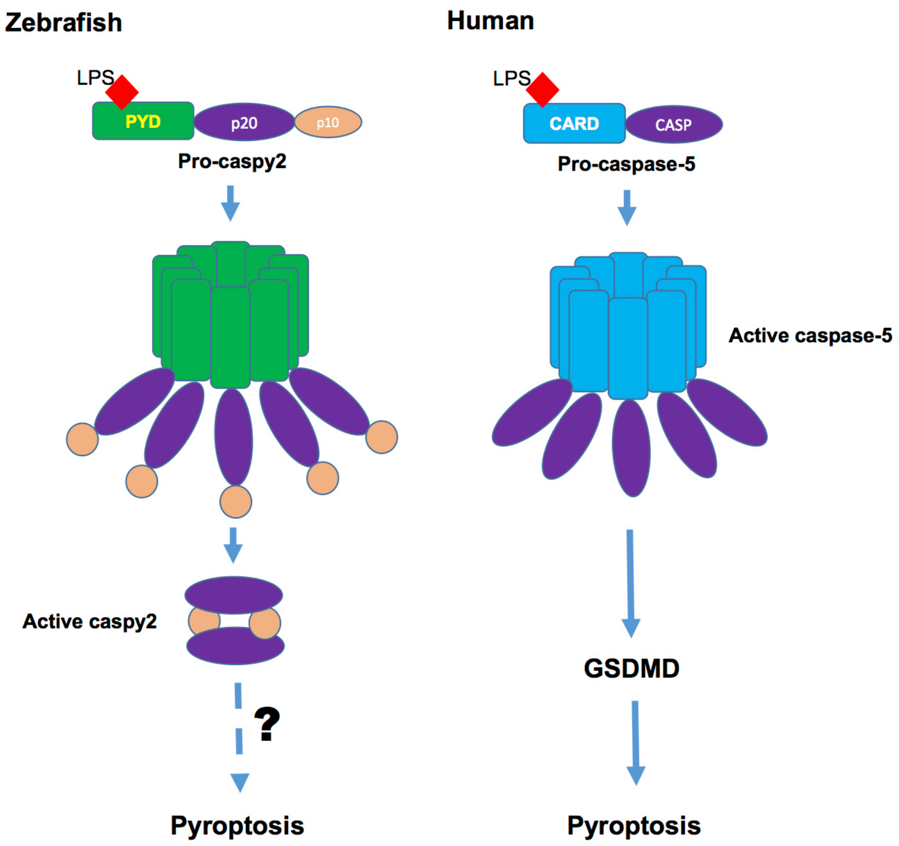

Supplementary Fig. 8. A schematic comparison of LPS binding by zebrafish caspy2 and human caspase-5 to mediate pyroptosis.

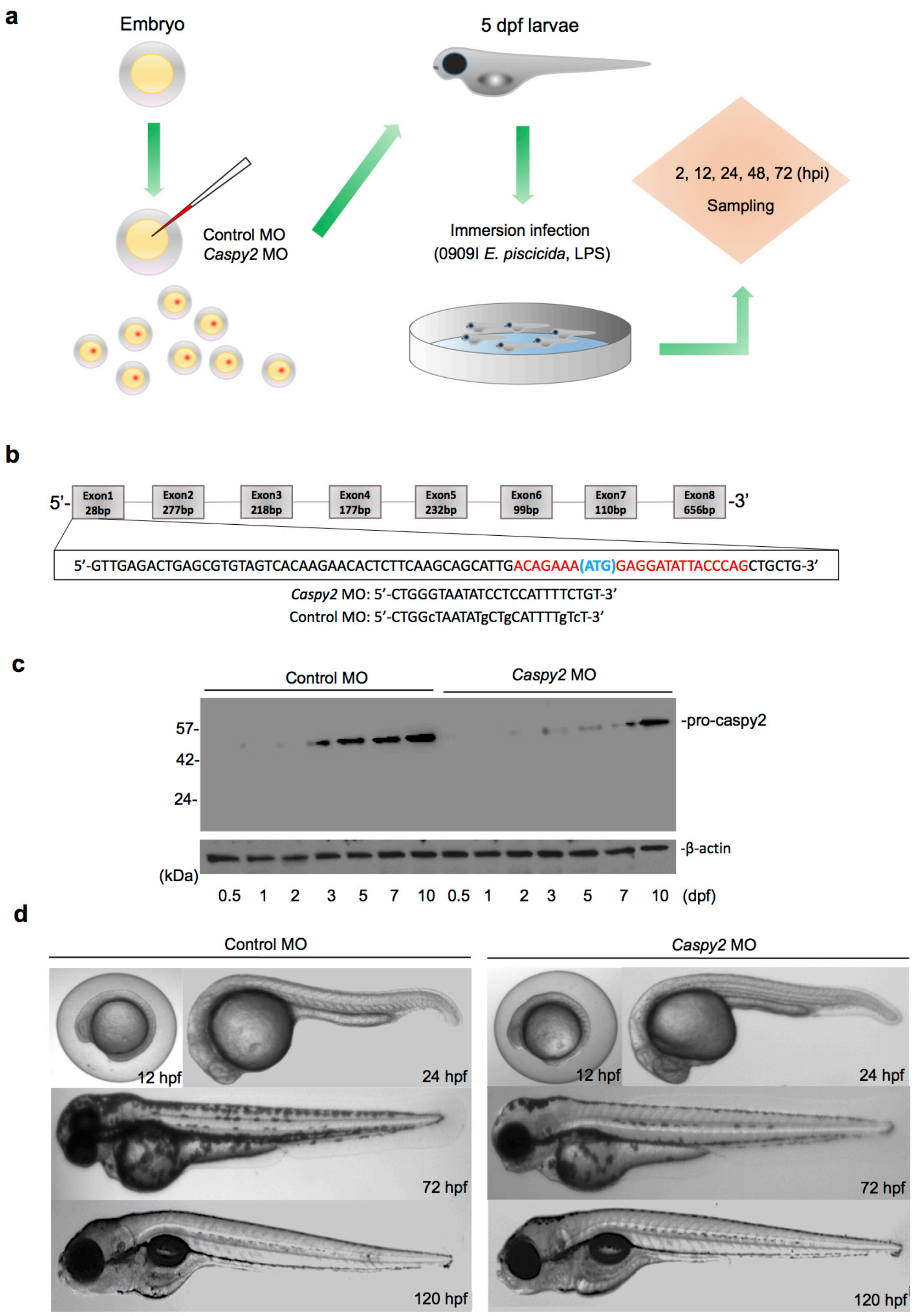

Supplementary Fig. 9. Representation of the zebrafish larvae immersion Infection Model. (a) The zebrafish microinjection and immersion infection procedures. (b) Generation of *caspy2*

morphant zebrafish by morpholino oligonucleotide (MO)-mediated knockdown. The upper diagram shows the region of *caspy2* targeted to block translation. (c and d) Analysis of the phenotypes of c*aspy2*-MO- and control-MO-injected larvae. (c) Immunoblotting of caspy2 expression in morphants and controls up to 5 days post fertilization (dpf). (d) Representative images of the phenotypes of *caspy2*-MO- and control-MO-injected zebrafish up to 120 h post fertilization (hpf). (c and d) Results are representative of at least three independent experiments.

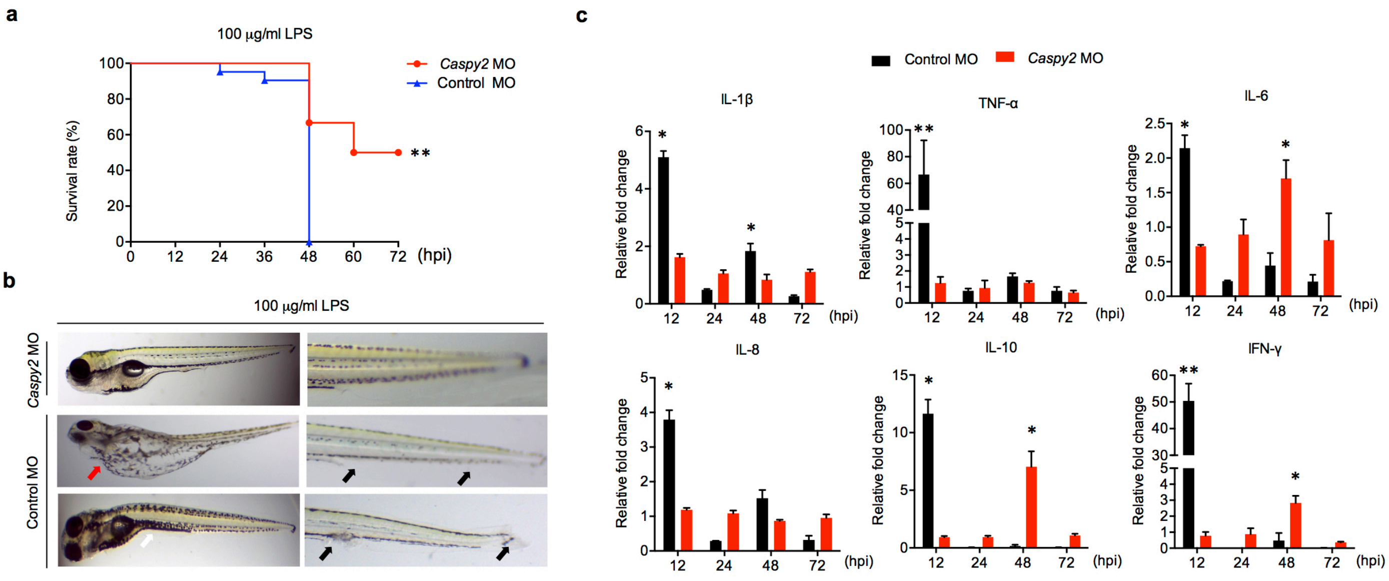

Supplementary Fig. 10. Caspy2 is critical for lethal sepsis in zebrafish larvae. (a-c) Control-morpholino oligonucleotide (MO) or *caspy2*-MO zebrafish larvae at 5 dpf were immersed in 100 µg/ml of LPS. (a) Survival was then monitored for 72 hpi. Results are representative of at least three independent experiments. ** $p < 0.01$ (log-rank). (b) Distinct pathologies associated with lethal dose of LPS. Pericardial edema (red arrow); Fin erosion and ulceration (black arrow); Protrusions on the trunk or tail regions (white arrow). Results are representative of at least three independent experiments. (c) RT-PCR analysis of the specified cytokine transcripts at the indicated time points in control- and *caspy2*-MO larvae. Each bar represents mean results SEM from 3-4 pools of 15 larvae. RT-PCR for each pool was carried out using technical duplicates. *$p < 0.05$, **$p < 0.01$ (*t*-test).

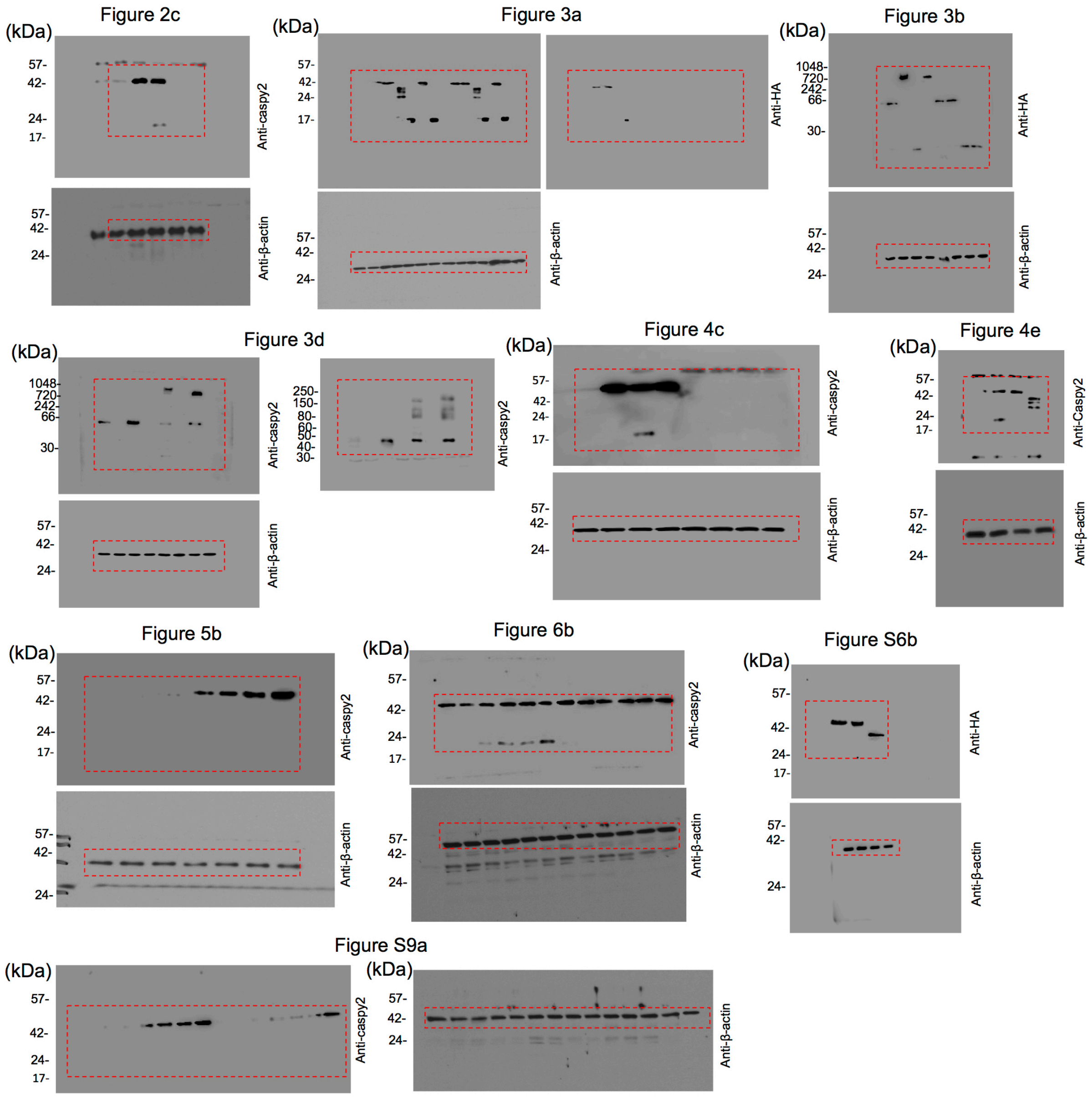

Supplementary Fig. 11. Full-length images of immunoblot analyses.

Supplementary Table 1. Primers for RT-PCR

Gene	(5' to 3')
β-actin F	ATGGATGAGGAAATCGCTGCC
β-actin R	CTCCCTGATGTCTGGGTCGTC
TNF-α F	AAGGAGAGTTGCCTTTACCG
TNF-α R	ATTGCCCTGGGTCTTATGG
IL-1β F	TGGACTTCGCAGCACAAAATG
IL-1β R	CACTTCACGCTCTTGGATGA
IL-6 F	TCAACTTCTCCAGCGTGATG
IL-6 R	TCTTTCCCTCTTTTCCTCCTG
IL-8 F	GTCGCTGCATTGAAACAGAA
IL-8 R	CTTAACCCATGGAGCAGAGG
IL-10 F	TCACGTCATGAACGAGATCC
IL-10 R	CCTCTTGCATTTCACCATATCC
IL-12 F	GAAACTCAACTGACCTCAACTG
IL-12 R	CTTTATCTGGCTTGACAATGTCTC
IFN-γ F	AAGATTCTCAGCTACATAATGCACACC
IFN-γ R	ATGCTCATCAGTAGATTCTGCTCAC
